# Clinical performance of the Lumipulse G NfL CSF assay in cases with AD and FTD

**DOI:** 10.1002/alz.087868

**Published:** 2025-01-09

**Authors:** Pablo Agüero, Raquel Téllez, Alicia Ruiz Gonzales, Sonia Wagner, Agustín Querejeta, Ignacio Mahillo, Andrea Ruiz, María José Sainz, Lucía Cremades‐Jimeno, Pascual Sanchez‐Juan, Estrella Gómez‐Tortosa

**Affiliations:** ^1^ Fundación Jiménez Díaz, Madrid, madrid Spain; ^2^ Alzheimer´s Centre Reina Sofía‐CIEN Foundation‐ISCIII, Madrid, madrid Spain; ^3^ Hospital Rey Juan Carlos, Móstoles Spain; ^4^ Reina Sofia Alzheimer Centre, CIEN Foundation, ISCIII, Madrid Spain

## Abstract

**Background:**

Neurofilament light chain (NfL) is a fluid biomarker of axonal damage reported to be elevated in cases with dementia, and particularly in FTD. In this study we evaluate the performance of a recently developed NfL assay to be analyzed through the Lumipulse chemiluminescent platform, which is frequently available in clinical settings for the study of AD core biomarkers.

**Method:**

We evaluated CSF NfL levels using the Lumipulse G600II platform (Fujirebio, Iberia) in 70 cases, including 33 patients with AD (supported by CSF biomarkers consistent with an A+T+(N)+ classification scheme), 26 with confirmed FTD (typical phenotype and CSF with a A‐T‐ profile), and 11 controls. NfL was also determined in plasma samples of the same cases by single molecule array (SIMOA) with a Quanterix assay. We analyzed the correlation of NfL as measured in these two samples by the two different platforms and their value to discriminate between AD and FTD cases.

**Result:**

NfL in CSF was higher in FTD (mean‐SD= 2417‐2326 pg/mL) than in AD cases (1144‐594, p=0.07) and controls (809‐327, p=0.03), with no differences between AD and controls. The same applied for plasma NfL with more remarkable statistical differences: FTD (mean‐SD= 37‐21) vs AD cases (19‐7, p>0.001) and vs controls (18‐7, p=0.007). There was a significant linear correlation between NfL values in CSF and plasma in all cases (Pearson R=0.84, p<0.001). By groups, the correlation was higher in FTD cases (R=0.83) and controls (R=0.89) than in AD cases (R=0.46). The best discrimination between FTD and AD cases for CSF NfL was obtained at 1671 pg/mL (AUC: 0.675, 59% sensitivity, 90.6% specificity), while levels of 1395 pg/mL obtained the best discrimination between FTD and controls (AUC: 0.777, sensitivity 64%, specificity 100%).

**Conclusion:**

The Lumipulse NfL CSF assay could be useful in clinical settings to improve discrimination among dementia subtypes and controls. Elevated NfL CSF levels are highly specific of FTD and correlate well with plasma levels. Furthermore, NfL levels may support an underlying neurodegenerative process in cases with ambiguous phenotype and an A‐T‐(N)‐ CSF profile.

